# Radiomics Based on DCE-MRI Improved Diagnostic Performance Compared to BI-RADS Analysis in Identifying Sclerosing Adenosis of the Breast

**DOI:** 10.3389/fonc.2022.888141

**Published:** 2022-05-12

**Authors:** Mei Ruan, Zhongxiang Ding, Yanna Shan, Shushu Pan, Chang Shao, Wen Xu, Tao Zhen, Peipei Pang, Qijun Shen

**Affiliations:** ^1^ Department of Radiology, Affiliated Hangzhou First People’s Hospital, Zhejiang University School of Medicine, Hangzhou, China; ^2^ Department of Pathology, Affiliated Hangzhou First People’s Hospital, Zhejiang University School of Medicine, Hangzhou, China; ^3^ Department of Pharmaceuticals Diagnosis, GE Healthcare, Hangzhou, China

**Keywords:** sclerosing adenosis, breast carcinoma, magnetic resonance imaging, radiomics, differential diagnosis

## Abstract

**Purpose:**

Sclerosing adenosis (SA) is a benign lesion that could mimic breast carcinoma and be evaluated as malignancy by Breast Imaging-Reporting and Data System (BI-RADS) analysis. We aimed to construct and validate the performance of radiomic model based on dynamic contrast-enhanced magnetic resonance imaging (DCE-MRI) compared to BI-RADS analysis to identify SA.

**Methods:**

Sixty-seven patients with invasive ductal carcinoma (IDC) and 58 patients with SA were included in this retrospective study from two institutions. The 125 patients were divided into a training cohort (n= 88) from institution I and a validation cohort from institution II (n=37). Dynamic contrast-enhanced sequences including one pre-contrast and five dynamic post-contrast series were obtained for all cases with different 3T scanners. Single-phase enhancement, multi-phase enhancement, and dynamic radiomic features were extracted from DCE-MRI. The least absolute shrinkage and selection operator (LASSO) logistic regression and cross-validation was performed to build the radscore of each single-phase enhancement and the final model combined multi-phase and dynamic radiomic features. The diagnostic performance of radiomics was evaluated by receiver operating characteristic (ROC) analysis and compared to the performance of BI-RADS analysis. The classification performance was tested using external validation.

**Results:**

In the training cohort, the AUCs of BI-RADS analysis were 0.71 (95%CI [0.60, 0.80]), 0.78 (95%CI [0.67, 0.86]), and 0.80 (95%CI [0.70, 0.88]), respectively. In single-phase analysis, the second enhanced phase radiomic signature achieved the highest AUC of 0.88 (95%CI [0.79, 0.94]) in distinguishing SA from IDC. Nine multi-phase radiomic features and two dynamic radiomic features showed the best predictive ability for final model building. The final model improved the AUC to 0.92 (95%CI [0.84, 0.97]), and showed statistically significant differences with BI-RADS analysis (*p*<0.05 for all). In the validation cohort, the AUC of the final model was 0.90 (95%CI [0.75, 0.97]), which was higher than all BI-RADS analyses and showed statistically significant differences with one of the BI-RADS analysis observers (*p* = 0.03).

**Conclusions:**

Radiomics based on DCE-MRI could show better diagnostic performance compared to BI-RADS analysis in differentiating SA from IDC, which may contribute to clinical diagnosis and treatment.

## 1 Introduction

Sclerosing adenosis (SA) is a common but poorly understood benign proliferative breast disease, which can mimic invasive carcinoma in both clinical palpation and imaging findings (1–4). It’s difficult to guide the formulation of clinical treatment strategies because SA and invasive carcinoma undergo different clinical treatments (1). The histopathologic examination may be necessary for a definite diagnosis of this condition. However, both biopsy and surgery are invasive and may lead to a series of complications, such as pain, infection, bleeding, local necrosis, psychological stress, etc. (5–8). Meanwhile, misdiagnosis may occur due to sampling errors in some biopsy cases. Accordingly, a preoperative, noninvasive, and clear approach to differentiate SA from invasive carcinoma is necessary and crucial to improve benefits for clinical management.

Previous studies have described the various radiological characteristics of SA (3, 4, 9, 10). SA may present mainly clustered microcalcifications, asymmetric focal density, or focal architectural distortion on mammography (3, 11). SA lesions can be detected as microlobulated, angulated, or spiculated mass on ultrasonography (4, 12). On magnetic resonance imaging (MRI), SA can be seen as an oval or irregular mass showing rapid early enhancement and delayed persistent or washout kinetics (3, 9, 13). The Breast Imaging-Reporting and Data System (BI-RADS) lexicon of the American College of Radiologists (ACR) was used to assess the risk of malignancy of breast lesions for further management (14). Due to the multiple and atypical imaging features, SA and invasive carcinoma could be classified into the same BI-RADS category, such as category 4, and even category 5. The area under the curve (AUC) of ultrasonography and mammography distinguishing between benign and malignant lesions was 0.55 and 0.50, respectively, by BI-RADS analysis as reported (15). It’s challenging for radiologists to accurately differentiate SA from invasive carcinoma through conventional imaging evaluation. Liang et al. (16) revealed that an ultrasound-based nomogram could be used as a supplement to distinguish malignant tumors from SA for precise biopsies. But their feature estimation relied on a subjective analysis with inevitable bias. It’s necessary to evaluate the differentiation by more objective parameters (3, 4, 17).

Recently, radiomics has shown promise in reflecting the relationships between radiological and pathological features more objectively through machine learning and statistical analysis methods. MRI-based radiomics has been widely studied and a number of studies have proven the ability of the classification and prognosis of breast carcinoma (18, 19). Whereas, the role of radiomics in differentiating SA from breast carcinoma is unclear. Furthermore, previous literature mainly focused on different single-static phases of enhanced images, and the consistency was controversial (19–23). The development of lesions was a dynamic process that cannot be fully reflected by single static characteristics. Multi-phase enhanced and dynamic radiomics has gained increasing attention and the roles remain to be further revealed (22, 24–26).

In this study, we hypothesized that radiomic analysis might identify the associations between the quantitative imaging features and the lesion pathophysiology. This study aims to establish a radiomic model that combined multi-phase enhancement and dynamic features on dynamic contrast‐enhanced magnetic resonance imaging (DCE-MRI) to evaluate their capacity in differentiating SA from breast carcinoma and to compare it with BI-RADS analysis. External validation was performed to assess the preoperative discrimination of the proposed model.

## 2 Materials and Methods

**Figure 1:**
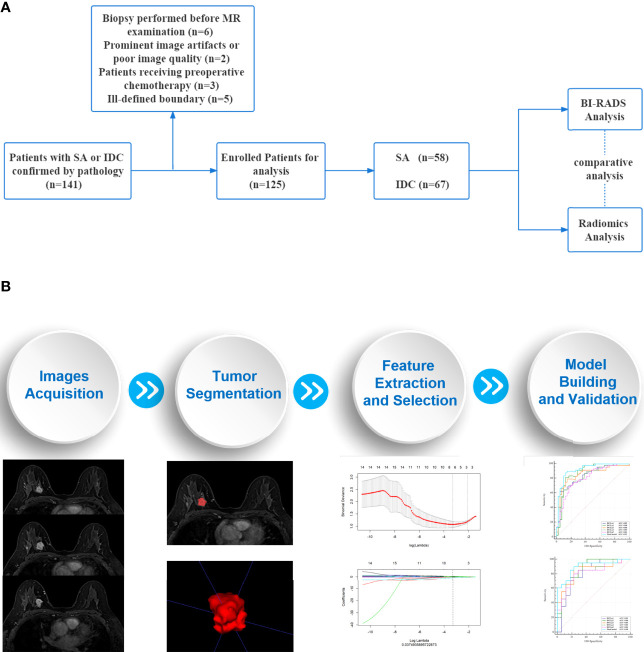
Workflow of the study. **(A)** An overview workflow of the present study. **(B)** The process of radiomics analysis was mainly composed of four parts: images acquisition, tumor segmentation, feature extraction and selection, model building and validation.

This retrospective study was approved by the Medical Ethics Committee of our institutions and the requirement for informed consent was waived. The workflow of the study is summarized in **Figure 1**.

### 2.1 Patient

Patients who underwent DCE-MRI examination between January 2015 and December 2019 were retrospectively collected in two institutions. The inclusion criteria for the study were as follows: (1) Patients were pathologically confirmed SA or invasive ductal carcinoma (IDC). (2) Patients received breast MRI examination within 2 weeks before surgery. (3) MRI scans were available for qualitative and radiomic analysis. (4) The boundary of the lesions was well-defined on MRI. (5) No previous chemotherapy or radiation therapy. (6) No biopsy or surgery before MRI examination.

### 2.2 Magnetic Resonance Imaging Protocols

MRI examination was performed with different scanners in two institutions (institution I: Magneton Verio, Siemens AG, 3T, Germany; institution II: Ingenia, Philips Healthcare, 3T, China). They applied the same protocol for dynamic contrast enhancement including one pre-contrast and five dynamic post-contrast series with fat-saturated T1-weighted dynamic sequences. Contrast material was injected into the elbow vein (0.1 mmol/kg of gadodiamide) and followed by a 20 mL saline flush at a rate of 2.0 mL/s. Contrast-enhanced images were acquired at 1, 2, 3, 4, and 5 minutes after contrast injection. The scanning parameters of the two institutions were as follows: (1) Institution I: TR 4.51, TE 1.61, flip angle 10°, slice thickness 1.0 mm, FOV 320× 320, image matrix 420 × 420; (2) Institution II: TR 4.2, TE 2.1, flip angle 12°, slice thickness 1.0 mm, FOV 339× 339, image matrix 407 × 407.

### 2.3 BI-RADS Analysis

According to the 5th edition ACR BI-RADS lexicon (14) on the standard protocol, MRI data were independently evaluated by three radiologists: Observer 1 (O1) with 9 years of experience, Observer 2 (O2) with 10 years of experience, and Observer 3 (O3) with 14 years of experience. The three radiologists were blinded to the clinical data and the pathological results. To assess the diagnostic accuracy of BI-RADS analysis, BI-RADS category 3 was considered as probably benign, and BI-RADS categories 4 and 5 were considered suspicious or highly suggestive of malignancy. The diagnostic performance of the three observers was analyzed.

### 2.4 Radiomic Analysis

#### 2.4.1 Image Processing and Tumor Segmentation

The original contrast-enhanced MRI images of enrolled patients were exported in Digital Imaging and Communication in Medicine (DICOM) format from the two institutions. MRI signal intensity standardization and gray-level quantization were applied to reduce the gray-level differences caused by the imaging procedure before delineation.

Two radiologists, Observer 4 (O4) with 4 years of experience and Observer 5 (O5) with 11 years of experience, who were blinded to the clinical data and pathological results, evaluated the contrast-enhanced MRI images using ITK-SNAP (Version 3.6) software for 3D manual segmentation. The volumes of interest (VOIs) were delineated along the inner margin of the tumor on each slice of the five enhanced phases images by the two observers, respectively. All pixels’ gray scales inside the VOIs were extracted for analysis.

#### 2.4.2 Feature Extraction and Selection

For each VOI, 396 radiomic features were extracted using the A.K. (Artificial Intelligent Kit, A.K., Version 3.2.2., GE Healthcare) software. The radiomic features were composed of six categories of parameters and classed as follows: Histogram features (n=42), texture features (n=10), gray level co-occurrence matrix (GLCM, n=144), gray level run length matrix (GLRLM, n=180), gray level size zone matrix (GLSZM, n=11), and morphological features (n=9). Five enhanced phases resulted in a total of 1980 features of each case for multi-phase analysis.

The interobserver agreement was assessed with the intraclass correlation coefficient (ICC) to evaluate the reliability and reproducibility. Features with ICCs higher than 0.75 were considered reliable and selected. A Spearman correlation analysis was performed to identify the highly correlated features. Features with a mean absolute correlation higher than 0.9 were considered redundant and eliminated. Then maximum relevance and minimum redundancy (mRMR) (27) were performed to eliminate the redundant and irrelevant features by the R package glmnet (version 3.3.2).

We defined the changes of radiomic features between each enhancement phase (Phase_x + 1_ – Phase_x_, for instance, Phase_2_- Phase_1_) as the dynamic radiomic features, which was consistent with the dynamic radiomic study in previous literature (25). The multi-phase enhancement features selected by mRMR were used to assess the dynamic radiomic analysis.

#### 2.4.3 Model Building and External Validation

The least absolute shrinkage and selection operator (LASSO) regression using 10-fold cross-validation was adopted to choose the optimized subset of features (28). We used the LASSO regression to build radiomic signatures based on each single-phase enhancement and a final model by combining multi-phase enhancement features and dynamic radiomic features. Features with non-zero coefficients were selected from the optimal features and were combined linearly to construct a radscore model.

### 2.5 Statistical Analysis

Statistical analysis was conducted by R software (version 3.5.1) and MedCalc (version 19.1). Statistical group comparisons of data were analyzed by A chi-square test or Fisher’s exact test (normal variables) and Mann-Whitney U test (continuous variables). *P* < 0.05 was considered statistically significant. The agreement between two radiologists was evaluated using interclass correlation coefficient (ICC) analysis, which was defined as good consistency between 0.75 and 1.00, fair consistency between 0.40 and 0.75, and poor consistency under 0.40. The correlation and collinearity of radiomic features were evaluated using the variance inflation factor (VIF) function. The radiomic models were tested using an independent testing set. The classification performance of BI-RADS analysis and radiomic analysis were respectively subjected to ROC analysis, by using sensitivity, specificity, and area under the ROC curve (AUC) to evaluate the classification efficacy. The comparison of ROC curves was performed by Delong’s test.

## 3 Results

### 3.1 Patients Characteristics

A total of 125 lesions from 125 patients (age range: 24-81 years; mean age: 49.97 ± 11.85 years) were recruited. The training cohort was comprised of patients from institution I (n =88). The external validation cohort was comprised of patients from institution II (n = 37). The pathological distribution was IDC in 47 patients, SA in 41 patients of institution I, and IDC in 20 patients, SA in 17 patients of institution II.

### 3.2 BI-RADS Analysis

**Figure 2:**
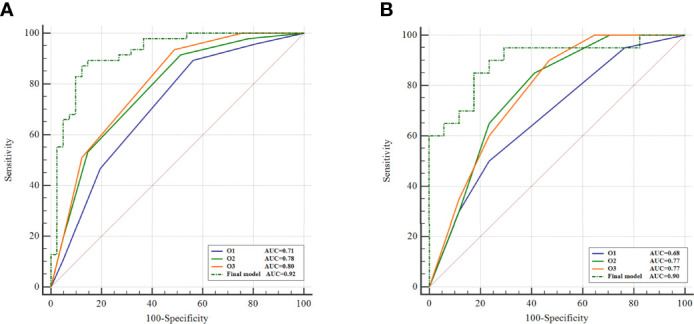
Comparison of BI-RADS analysis and radiomics. ROC curves of BI-RADS analysis and the final model of radiomics on training cohort **(A)** and external validation cohort **(B)**.

The AUC values of the three observers (O1, O2, and O3) were 0.71 (95%CI [0.60, 0.80] *p*<0.001), 0.78 (95%CI [0.67, 0.86] *p*<0.001), and 0.80 (95%CI [0.70, 0.88) *p*<0.001] in the training cohort, respectively (**Table 1**; **Figure 2**). There were statistically significant differences between O1 and O2, O3 (O1 vs O2, *p* = 0.001; O1 vs O3, *p* < 0.001), and there was no statistically significant difference between O2 and O3 (*p*=0.116). In the validation cohort, the AUC values of the three observers were 0.68 (95%CI [0.50, 0.82] *p* = 0.042), 0.77 (95%CI [0.61, 0.89] *p* < 0.001) and 0.77 (95%CI [0.61, 0.89] *p* < 0.001), respectively (**Table 1**; **Figure 2**). There were statistically significant differences between O1 and O2, O3 (O1 vs O2, *p* < 0.001; O1 vs O3, *p* < 0.001), and there was no statistically significant difference between O2 and O3 (*p* = 0. 94).

**Table 1:** Performance of the three observers of BI-RADS analysis.

Observer	Training cohort				Validation cohort			
	SEN	SPEC	AUC (95%CI)	*p* value	SEN	SPEC	AUC (95%CI)	*p* value
O1	0.89	0.44	0.71 (0.60-0.80)	<0.001	0.50	0.77	0.68 (0.50-0.82)	0.042
O2	0.92	0.49	0.78 (0.67-0.86)	<0.001	0.85	0.59	0.77 (0.61~0.89)	0.001
O3	0.94	0.51	0.80 (0.70-0.88)	<0.001	0.90	0.53	0.77 (0.61-0.89)	0.001

O1, 2, 3 BI-RADS analysis of Observer 1, 2, 3; AUC, area under the ROC curve; SEN, sensitivity; SPEC, specificity.

### 3.3 Radiomic Analysis

Based on the result of reproducibility analysis by two radiologists (O4, O5), 1794 out of 1980 (90.6%) radiomic features had good consistency (ICC ≥ 0.75). The numbers of features with fair consistency (0.75 > ICC ≥ 0.40) and poor consistency (ICC <0.4) were 99 (5.0%) and 87 (4.4%), respectively. Features with an ICC ≥ 0.75 were considered robust and were maintained for further processing. We randomly selected one of the groups of data for radiomic analysis on account of the good level of consistency. Then, mRMR was applied to eliminate the redundant and irrelevant features. In this study, only 20 features were retained by mRMR. The LASSO classifier was used to select the optimal radiomic feature subset to build a radscore.

**Figure 3:**
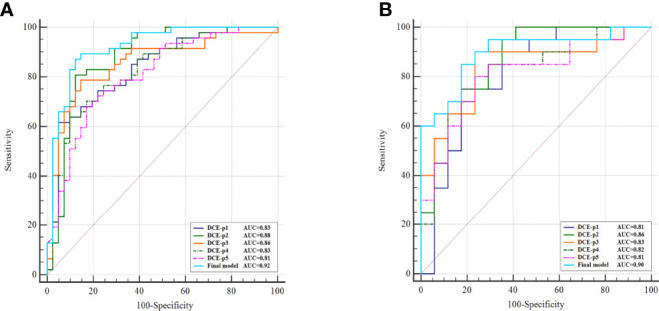
Comparison of single phase enhancement and the final model of radiomics. ROC curves of single phase enhancement and the final model on training cohort **(A)** and external validation cohort **(B)**.

In the training cohort, the AUCs ranged from 0.81 to 0.88 for the five single phase enhancement of radiomic analysis, in which DCE-phase2 obtained the best performance with an AUC of 0.88 (95% CI [0.79, 0.94] *p*<0.001) (**Table 2**; **Figure 3**).

**Table 2:** Performance of single phase enhancement and the final model of radiomics analysis.

Model	Training cohort				Validation cohort			
	SEN	SPEC	AUC (95%CI)	*p* value	SEN	SPEC	AUC (95%CI)	*p* value
DCE-p1	0.62	0.95	0.83 (0.74-0.91)	<0.001	0.75	0.82	0.81 (0.65-0.92)	<0.001
DCE-p2	0.81	0.88	0.88 (0.79-0.94)	<0.001	0.95	0.65	0.86 (0.71-0.95)	<0.001
DCE-p3	0.79	0.85	0.86 (0.77-0.92)	<0.001	0.90	0.76	0.83 (0.68-0.94)	<0.001
DCE-p4	0.64	0.90	0.83 (0.73-0.90)	<0.001	0.70	0.88	0.82 (0.66-0.93)	<0.001
DCE-p5	0.68	0.83	0.81 (0.71-0.88)	<0.001	0.80	0.77	0.81 (0.65~0.92)	<0.001
Final model	0.87	0.88	0.92 (0.84-0.97)	<0.001	0.85	0.82	0.90 (0.75-0.97)	<0.001

DCE-p dynamic contrast enhanced phase.

A total of 11 optimal features, nine from the multi-phase enhancement (one was from DCE-phase1, six were from DCE-phase2 and two were from DCE-phase3), and two from dynamic radiomic features, showed the best predictive ability for final model building with AUC value of 0.92 (95%CI [0.84, 0.97] *p* < 0.001) (**Table 2**). There was no collinearity among the 11 features after verification by the VIF function. Details of the correlation between the 11 optimal features and radscore formula are described in Supplementary materials.

The diagnostic performance of the single-phase enhancement and the final model of radiomics was validated using external validation data collected from institution II, with the AUCs ranged from 0.81 to 0.86 for the five single-phase enhancement of radiomic analysis. And the DCE-phase2 obtained the highest AUC value of 0.86 (95%CI [0.71, 0.95] *p* < 0.001). The final model displayed AUC of 0.90 (95%CI [0.75, 0.97] *p* < 0.001).

**Figure 4:**
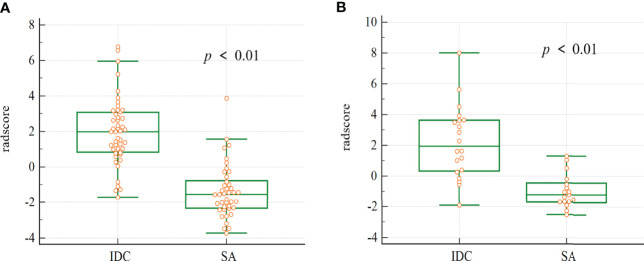
The box plot of the radscore in the final model. Mann-whitney analysis of radscore for distinguishing SA from IDC in the training cohort **(A)** and validation cohort **(B)** (*p* < 0.01).

There were statistically significant differences in the radscore values for both the training cohort and validation cohort of SA and IDC (**Figure 4**).

### 3.4 Comparative Analysis

AUC of the final model was higher in differentiating SA from IDC compared to both BI-RADS analysis and single-phase enhancement in both training and validation cohorts, and all three observers of BI-RADS analysis showed statistically significant differences with the final model in the training (*p*<0.05 for all) (**Figure 2**). In the validation cohort, the final model showed a statistically significant difference with O1 of the BI-RADS analysis observers (*p* = 0.03). Details of the comparison of BI-RADS analysis and the final model of radiomics are described in Supplementary materials.

**Figure 5:**
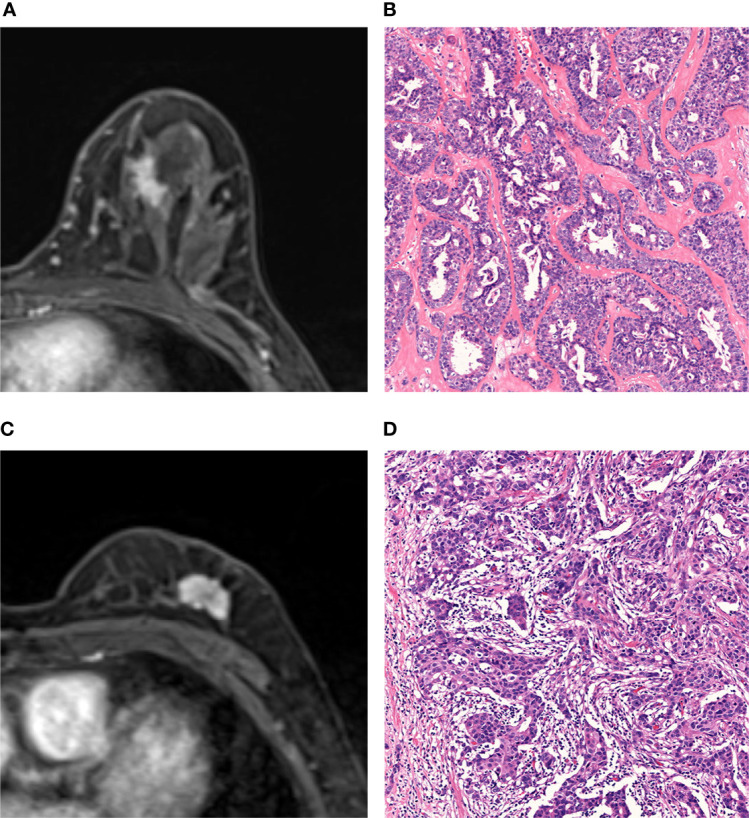
MRI and histopathologic findings of two cases of SA and IDC. **(A, B)** MRI and histopathologic findings of a 47-year-old woman with SA. The DCE-MRI image showed an irregularly shaped mass with spiculated margin and heterogeneously enhancement **(A)**. The lesion was classified as malignant by BI-RADS analysis and benign by radiomic analysis. Histopathological examination proves SA (Hematoxylin-eosin staining; original magnification×100) **(B)**. **(C, D)** MRI and histopathologic findings of a 66-year-old woman with IDC. The DCE-MRI image showed an irregularly shaped mass with lobulated margin and heterogeneously enhancement **(C)**. The lesion was classified as malignant by both BI-RADS analysis and radiomic analysis. The histopathologic result was IDC (Hematoxylin-eosin staining, original magnification×100) **(D)**.

**Figure 5** shows two cases with SA and IDC, respectively, and indicates that the final model of radiomics can differentiate SA from IDC when the lesions present similar MRI findings.

## 4 Discussion

Our study established and validated a final model which incorporated multi-phase enhancement and dynamic radiomic features on DCE-MRI for differentiation between SA and IDC. The final model showed better diagnostic efficacy than either the BI-RADS analysis or radiomic analysis of single-phase alone, which indicated the superiority of the multi-phase enhanced scanning and kinetic parameters in the disease identification.

SA is a benign but complex lesion characterized pathologically with the proliferation of the epithelial, myoepithelial, and basement membrane (5, 17). SA can form adenosis tumors and may be confused with invasive carcinoma because of an irregular pattern with involvement of the adipose tissue, fibromammary tissue, and pseudo perineural invasion on routine hematoxylin-eosin staining. Immunohistochemistry of the myoepithelium is requisite for a definite diagnosis of this condition. A previous study described MRI features of SA of the breast with correlation to the pathology and showed that SA component was associated with masses with indeterminate or suspicious kinetics (13). These may be the plausible reasons why SA could mimic invasive carcinoma on clinical and imaging presentation. Although research has reported that SA may convey an approximate doubling of breast cancer risk as a single feature, the presence of sclerosing adenosis alone in a core biopsy does not require surgical excision (29, 30). Close clinical follow-up or routine imaging is recommended (1, 7, 31). Accurately distinguishing SA from invasive carcinoma *via* a non-invasive, preoperative method is crucial to help avoid unnecessary biopsy and surgery for both patients and clinicians.

The studies of differentiating SA from invasive carcinoma on MRI were scarce. The BI-RADS atlas provided standardized imaging terminology to assess the risk of malignancy while the imaging evaluation was based on subjective observation by the naked eye. Liu et al. (4) found that the BI-RADS atlas could be a powerful tool in demonstrating the SA lesion, and in differentiating SA from IDC lesions on ultrasonography. Nevertheless, it’s regrettable that the study didn’t indicate the diagnostic efficacy and there was no assessment of inter-group consistency. Liang et al. (16) developed an ultrasound-based nomogram for distinguishing malignant tumors from nodular SA and demonstrated that the nomogram could build a precise sequence of biopsies when multiple nodular SA and malignant masses were classified into the same BI-RADS category. Both of the above studies were estimated by relying on a subjective analysis with inevitable bias.

In our study, the AUCs of three observers ranged from 0.68 to 0.80 and the diagnostic capacity of BI-RADS analysis based on experiences showed inconsistency. There were statistical differences between observers with varying experiences (O1 vs O2, O1 vs O3, *p* < 0.01), indicating that conventional image evaluation might be influenced by subjective experience. Compared with the BI-RADS analysis based on qualitative assessment, the advantage of the fully quantitative radiomic analysis is reflected in the consistency between observers of different experiences. 90.6% of radiomic features had good consistency (ICC ≥ 0.75) despite significant differences in experience between the two radiologists who performed radiomic analysis. Even junior physicians can accurately delineate tumors on MR images, and distinguish SA from invasive carcinoma preliminarily by radiomic analysis. Texture parameters, GLCM parameters, and GLRLM parameters contributed to the final model construction, which indicated that the heterogeneity of lesions was more sensitive in differentiation. Radiomic features represented underlying histologic characteristics that could not be acquired by the observer’s naked eye. In addition, our study also showed the final radiomic model was superior to BI-RADS analysis.

Dynamic contrast-enhanced scans have been widely used in breast radiomic studies, however, extracting post-contrast images at which time points was controversial (19). Ahmed et al. (32) found that texture features showed differences among different phases after enhancement. Significant differences were mainly seen at 1-3 minutes post-contrast administration. Karahaliou et al. (24) and Fan et al. (22) analyzed the images of two phases after enhancement and showed different results. The contrast enhancement performance was related to the abnormal tumor angiogenesis. These malignant lesion vessels tend to be large, leaky, and typically showed intense enhancement with rapid uptake and washout of contrast, while benign lesions and normal tissues were slower and less intense enhancement (33). Thus, the intensity of lesion enhancement on MRI at post-contrast 2 min was considered the most critical in conventional image assessment (33, 34). In our study, DCE-phase2 presented the best diagnostic performance among single-phase enhancement analyses, which was consistent with the previous study.

DCE-MRI can provide tumor kinetic characteristics by generating pharmacokinetic maps of contrast agents. Previous research had shown that kinetic characteristics improved the diagnostic performance of enhancement sequences (23, 35–37). Jiang et al. (23) and Chai et al. (37) both showed the diagnostic performance of the combination of kinetic and radiomic features was superior to radiomic features alone, but the two studies did not perform external validation to confirm the generalization under different scanners. In addition, Chai et al. (37) only analyzed the single-layer image of lesions. Previously we established and validated a nomogram model combined radiomics and kinetic curve pattern to detect metastatic axillary lymph nodes in patients with invasive breast cancer, which showed a better performance than the radiomic model or the kinetic curve pattern alone (38). However, the kinetic curve pattern was evaluated by naked eyes in routine assessment, which caused inevitably inconsistency due to subjectivity. To a certain extent, different scanning protocols may affect the pattern of the kinetic curve. Consequently, we improved it by conducting quantitative analysis on the kinetic changes between adjacent phases and the AUC of the final model reached 0.90 with the dynamic radiomic features.

There were some limitations in the current study that still need to be further investigated: (1) This study was a retrospective analysis, and the number of SA cases was limited. (2) No comparison or combination with DWI analysis was performed in this study. (3) The efficacy of clinical factors was not evaluated. (4) In the dynamic radiomic analysis, we only calculated the primary kinetic change of the two adjacent phases. More time-related features with a large sample are expected to verify the conclusions in further studies.

## 5 Conclusion

Our study showed that a final model integrated multi-phase enhancement and dynamic radiomic features extracted from DCE-MRI could show better diagnostic performance compared to BI-RADS analysis in distinguishing SA from IDC. Radiomics based on DCE-MRI might help clinicians to make more appropriate management for each patient.

## Data Availability Statement

The original contributions presented in the study are included in the article/**Supplementary Material**. Further inquiries can be directed to the corresponding author.

## Ethics Statement 

The studies involving human participants were reviewed and approved by Affiliated Hangzhou First People’s Hospital, Zhejiang University School of Medicine. Written informed consent for participation was not required for this study in accordance with the national legislation and the institutional requirements.

## Author Contributions

QS and ZD put forward the concept of the study, designed the study. ZD, YS, SP, WX, and TZ contributed to the data acquisition, analysis, and interpretation. PP and WX carried out the data analysis. MR contributed to prepare the manuscript and the statistical analysis. QS reviewed the manuscript. CS provided pathological analysis. All authors read and approved the final manuscript.

## Funding

This study was granted by the Medical Health Science and Technology Commission of Zhejiang Province, China (No. 2021KY240), the Natural Science Foundation of Zhejiang Province, China (No. LSY19H180009), and Clinical Science Foundation of ZheJiang University, China (No. YYJJ2019Z06), the Science and Technology Project of the development of Hangzhou Biomedicine and Health, China (No. 2021WJCY028).

## Conflict of Interest

Author PP is an employee of GE Healthcare.

The remaining authors declare that the research was conducted in the absence of any commercial or financial relationships that could be construed as a potential conflict of interest.

## Publisher’s Note

All claims expressed in this article are solely those of the authors and do not necessarily represent those of their affiliated organizations, or those of the publisher, the editors and the reviewers. Any product that may be evaluated in this article, or claim that may be made by its manufacturer, is not guaranteed or endorsed by the publisher.
