# Supplementary material for: Radiomics Based on DCE-MRI Improved Diagnostic Performance Compared to BI-RADS Analysis in Identifying Sclerosing Adenosis of the Breast
Source: Front Oncol. 2022 May 12;12:888141. doi: 10.3389/fonc.2022.888141 (PMC9133496; doi:10.3389/fonc.2022.888141)
Supplement: Supplementary file 1 [file DataSheet_1.docx]

**Supplementary Materials**

**1 Correlation of eleven features**

The correlation coefficient between each pair is less than 0.5 (Figure 1), that means there is no collinearity between the five features.


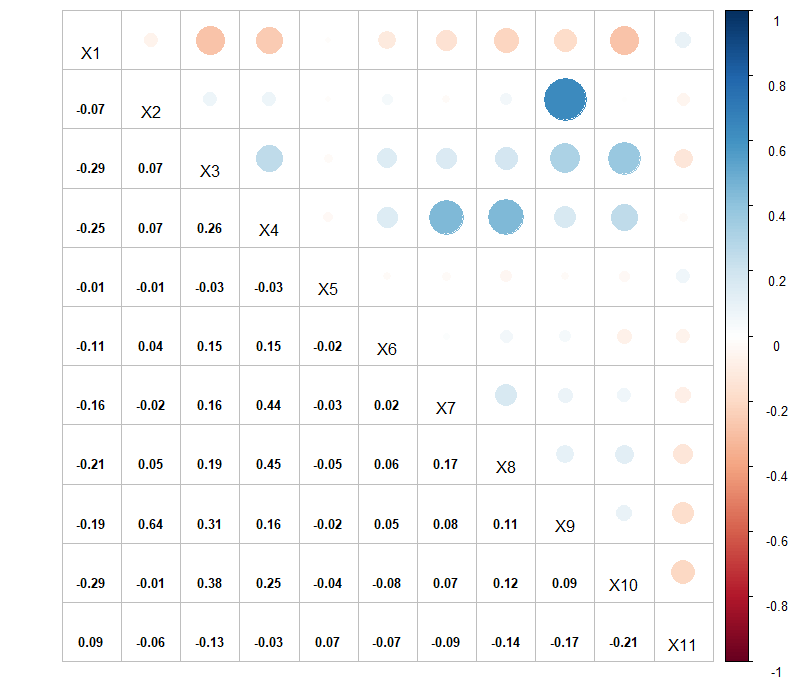


**Figure 1. Correlation of the features.**

The x1-11 represents the eleven features: DCE-p2_RLN_a45o7，DCE-p1_GLCME_ADo7SD, DCE-p2_CS_ADo7, DCE-p2_GLCME_a0o4, DCE-p2_LRLGLE_a45o4, DCE-p3_GLCME_a0o7, DCE-p3_GLCME_a135o7, DCE-p2_LRE_a0o1, DCE-p2_C_ADo7SD, CS_ADo7 of DCE-p_2-1_, GLCME_a135o7 of DCE-p_3-2_, the correlation coefficient between each pair is less than 0.5. There is no collinearity after verifying the correlation of the eleven features.

2 The eleven parameters of the final model and their coefficients

Table 1 The eleven parameters of the final model and their coefficients

|  | Feature class | Significant features | coefficient |
| --- | --- | --- | --- |
| Static radiomics features | Texture Parameters | DCE-p2_ CS_AD_o7 | -1.643 |
|  |  | DCE-p2_ C_AD_o7_SD | 0.496 |
|  | GLCM Parameters | DCE-p1_ GLCME_AD_o7_SD | 0.345 |
|  |  | DCE-p2_ GLCME_a0_o4 | -1.347 |
|  |  | DCE-p3_GLCME_ a0_o7 | 0.36 |
|  |  | DCE-p3_GLCME_ a135_o7 | -2.516 |
|  | GLRLM Parameters | DCE-p2_ RLN_a45_o7 | 1.147 |
|  |  | DCE-p2_ LRLGLE_a45_o4 | 0.08 |
|  |  | DCE-p2_ LRE_a0_o1 | -1.079 |
| Dynamic radiomics features | Texture Parameters | DCE-p_2-1_ CS_AD_o7 | -1.316 |
|  | GLCM Parameters | DCE-p_3-2_ GLCME_a135_o7 | -0.627 |

a, angle; o, offset; AD, AllDirection; CS, ClusterShade.

3 The radscore formula of the final model

$$\boldsymbol{Radscore=1.147*DCE\_p2}\boldsymbol{\_RLN\_a45o7+0.345*DCE\_p1\_GLCME\_ADo7SD-1.643*}\boldsymbol{DCE\_p2\_CS}\boldsymbol{\_ADo7}\boldsymbol{-1.347*DCE\_p2\_GLCME\_a0o4+}\boldsymbol{0.08*DCE\_p2\_LRLGLE}\boldsymbol{\_a45o4}\mathbf{+}\boldsymbol{0.36*DCE\_p3\_GLCME}\boldsymbol{\_a0o7}\boldsymbol{-2.516*DCE\_p3\_GLCME}\boldsymbol{\_a135o7}\boldsymbol{-1.079*DCE\_p2\_LRE}\boldsymbol{\_a0o1}\boldsymbol{+0.496*DCE\_p2\_C}\boldsymbol{\_ADo7SD}\boldsymbol{- 1.316*DCE\_p(2-1) CS}\boldsymbol{\_ADo7}\boldsymbol{-0.627*DCE\_p(3-2) GLCME}\boldsymbol{\_a135o7}\mathbf{-0.226}$$

**4 Comparison of BI-RADS and Radscore**

The Delong’s test was performed to compare the AUCs of BI-RADS and Radscore. The Radscore had a significant improvement than the three observers of BI-RADS analysis in the training cohort (p<0.05). The improvement of the Radscore was significant compared to O1 of BI-RADS analysis in the validation cohort (p<0.05). Detailed in Table S2and S3.

Table 2 Comparison of ROC curves of BI-RADS and Radscore in training cohort

| Final model ~ O1 | |
| --- | --- |
| Difference between areas | 0.210 |
| Standard Error ^a^ | 0.0625 |
| 95% Confidence Interval | 0.0877 to 0.333 |
| z statistic | 3.365 |
| Significance level | P = 0.0008 |
| Final model ~ O2 | |
| Difference between areas | 0.145 |
| Standard Error ^a^ | 0.0577 |
| 95% Confidence Interval | 0.0317 to 0.258 |
| z statistic | 2.509 |
| Significance level | P = 0.0121 |
| Final model ~ O3 | |
| Difference between areas | 0.122 |
| Standard Error ^a^ | 0.0556 |
| 95% Confidence Interval | 0.0130 to 0.231 |
| z statistic | 2.193 |
| Significance level | P = 0.0283 |
| O1 ~ O2 | |
| Difference between areas | 0.0654 |
| Standard Error ^a^ | 0.0198 |
| 95% Confidence Interval | 0.0266 to 0.104 |
| z statistic | 3.300 |
| Significance level | P = 0.0010 |
| O1 ~ O3 | |
| Difference between areas | 0.0882 |
| Standard Error ^a^ | 0.0231 |
| 95% Confidence Interval | 0.0430 to 0.133 |
| z statistic | 3.825 |
| Significance level | P = 0.0001 |
| O2 ~ O3 | |
| Difference between areas | 0.0228 |
| Standard Error ^a^ | 0.0145 |
| 95% Confidence Interval | -0.00563 to 0.0513 |
| z statistic | 1.572 |
| Significance level | P = 0.1159 |

Table 3 Comparison of ROC curves of BI-RADS and Radscore in validation cohort

| Final model ~ O1 | |
| --- | --- |
| Difference between areas | 0.219 |
| Standard Error ^a^ | 0.102 |
| 95% Confidence Interval | 0.0184 to 0.420 |
| z statistic | 2.140 |
| Significance level | P = 0.0323 |
| Final model ~ O2 | |
| Difference between areas | 0.125 |
| Standard Error ^a^ | 0.0924 |
| 95% Confidence Interval | -0.0560 to 0.306 |
| z statistic | 1.353 |
| Significance level | P = 0.1760 |
| Final model ~O3 | |
| Difference between areas | 0.124 |
| Standard Error ^a^ | 0.0937 |
| 95% Confidence Interval | -0.0601 to 0.307 |
| z statistic | 1.319 |
| Significance level | P = 0.1873 |
| O1 ~ O2 | |
| Difference between areas | 0.0941 |
| Standard Error ^a^ | 0.0315 |
| 95% Confidence Interval | 0.0324 to 0.156 |
| z statistic | 2.990 |
| Significance level | P = 0.0028 |
| O1 ~ O3 | |
| Difference between areas | 0.0956 |
| Standard Error ^a^ | 0.0324 |
| 95% Confidence Interval | 0.0320 to 0.159 |
| z statistic | 2.948 |
| Significance level | P = 0.0032 |
| O2 ~ O3 | |
| Difference between areas | 0.00147 |
| Standard Error ^a^ | 0.0196 |
| 95% Confidence Interval | -0.0369 to 0.0399 |
| z statistic | 0.0751 |
| Significance level | P = 0.9401 |

O1, 2, 3 BI-RADS analysis of Observer 1, 2, 3
